# Adenosine A_2A_ Receptors Measured with [^11^C]TMSX PET in the Striata of Parkinson's Disease Patients

**DOI:** 10.1371/journal.pone.0017338

**Published:** 2011-02-28

**Authors:** Masahiro Mishina, Kiichi Ishiwata, Mika Naganawa, Yuichi Kimura, Shin Kitamura, Masahiko Suzuki, Masaya Hashimoto, Kenji Ishibashi, Keiichi Oda, Muneyuki Sakata, Makoto Hamamoto, Shiro Kobayashi, Yasuo Katayama, Kenji Ishii

**Affiliations:** 1 Positron Medical Center, Tokyo Metropolitan Institute of Gerontology, Itabashi-ku, Tokyo, Japan; 2 The Second Department of Internal Medicine, Nippon Medical School, Bunkyo-ku, Tokyo, Japan; 3 Department of Neurology, Nippon Medical School Chiba Hokusoh Hospital, Inzai-shi, Chiba, Japan; 4 Department of Diagnostic Radiology, PET Center, Yale University, New Haven, Connecticut, United States of America; 5 Biophysics Group, Molecular Imaging Center, National Institute of Radiological Sciences, Chiba, Japan; 6 Department of Internal Medicine, Nippon Medical School Musashi Kosugi Hospital, Kawasaki, Kanagawa, Japan; 7 Department of Neurology, The Jikei University School of Medicine, Minato-ku, Tokyo, Japan; 8 Department of Neurology and Neurological Science Graduate School, Tokyo Medical and Dental University, Tokyo, Japan; 9 Department of Neurosurgery, Nippon Medical School Chiba Hokusoh Hospital, Inzai-shi, Chiba, Japan; Universidade Federal do Rio de Janeiro, Brazil

## Abstract

Adenosine A_2A_ receptors (A2ARs) are thought to interact negatively with the dopamine D_2_ receptor (D2R), so selective A2AR antagonists have attracted attention as novel treatments for Parkinson's disease (PD). However, no information about the receptor in living patients with PD is available. The purpose of this study was to investigate the relationship between A2ARs and the dopaminergic system in the striata of drug-naïve PD patients and PD patients with dyskinesia, and alteration of these receptors after antiparkinsonian therapy. We measured binding ability of striatal A2ARs using positron emission tomography (PET) with [7-methyl-^11^C]-(*E*)-8-(3,4,5-trimethoxystyryl)-1,3,7-trimethylxanthine ([^11^C]TMSX) in nine drug-naïve patients with PD, seven PD patients with mild dyskinesia and six elderly control subjects using PET. The patients and eight normal control subjects were also examined for binding ability of dopamine transporters and D2Rs. Seven of the drug-naïve patients underwent a second series of PET scans following therapy. We found that the distribution volume ratio of A2ARs in the putamen were larger in the dyskinesic patients than in the control subjects (p<0.05, Tukey-Kramer *post hoc* test). In the drug-naïve patients, the binding ability of the A2ARs in the putamen, but not in the head of caudate nucleus, was significantly lower on the more affected side than on the less affected side (p<0.05, paired *t*-test). In addition, the A2ARs were significantly increased after antiparkinsonian therapy in the bilateral putamen of the drug-naïve patients (p<0.05, paired *t*-test) but not in the bilateral head of caudate nucleus. Our study demonstrated that the A2ARs in the putamen were increased in the PD patients with dyskinesia, and also suggest that the A2ARs in the putamen compensate for the asymmetrical decrease of dopamine in drug-naïve PD patients and that antiparkinsonian therapy increases the A2ARs in the putamen. The A2ARs may play an important role in regulation of parkinsonism in PD.

## Introduction

Parkinson's disease (PD) is a progressive degenerative neurological disorder characterized clinically by resting tremor, bradykinesia, cogwheel rigidity, and postural instability [1]. These symptoms result primarily from the loss of dopaminergic neurons in the substantia nigra and can be reduced by levodopa, which replenishes dopamine, and dopamine agonists.

Adenosine is as an endogenous modulator of synaptic function in the central nervous system [2], and its effects are mediated by at least four receptor subtypes: A_1_, A_2A_, A_2B_, and A_3_ [3]. Adenosine A_1_ receptors (A1Rs) are widely distributed throughout the entire brain, while adenosine A_2A_ receptors (A2ARs) are enriched in the basal ganglia [4], [5]. The A2AR is known to stimulate adenylyl cyclase and interacts with the dopamine D_2_ receptor (D2R) negatively at the level of second messengers and beyond [5]. The A1R is known to inhibit adenylyl cyclase.

Recently, A2AR antagonists have attracted attention as potential non-dopaminergic therapies for PD. For example, caffeine is a nonselective adenosine receptor antagonist and is known to reduce the risk of developing PD [6], [7]. In addition, theophylline, which is also a nonselective adenosine receptor antagonist, was expected to be a promising agent for the treatment of PD [8]. However, findings from clinical trials of both caffeine and theophylline have been unimpressive [9], [10]. The selective A2AR antagonists was developed as novel nondopaminergic agents for PD [11] and provides antiparkinsonian benefit without causing or worsening dyskinesia, which is one of the most inconvenient side effects of dopaminergic therapy [12]. A postmortem study reported that the density of A2AR binding sites in PD was comparable to that found in the normal subjects, while the density in the basal ganglia was lower in patients with Huntington's chorea than in normal subjects [13]. However, another study using *in situ* hybridization and autoradiography suggested that A2ARs were involved in the development of dyskinesia following long-term levodopa therapy in PD [14]. Therefore, A2ARs may be involved in the appearance of the side effects of antiparkinsonian agents. Although the A2AR has attracted much attention in PD therapies, clinical evidence describing A2ARs in living PD patients, such as drug-naïve patients, is lacking.

**Figure 1:**
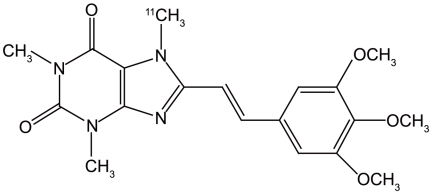
Structure of [^11^C]TMSX.

We developed PET ligands for mapping adenosine receptors, and we successfully visualized A1Rs with [1-methyl-^11^C]8-dicyclopropylmethyl-1-methyl-3-propylxanthine ([^11^C]MPDX) [15], [16] and A2ARs with [7-methyl-^11^C]-(*E*)-8-(3,4,5-trimethoxystyryl)-1,3,7-trimethylxanthine ([^11^C]TMSX, Fig. 1) [17], [18], [19], [20]. [^11^C]TMSX has a xanthine structure and is an analog of istradefylline. In addition, the specific binding of [^11^C]TMSX to A2ARs was confirmed with a theophylline challenge [21]. We performed test-retest studies and optimized the kinetics for [^11^C]TMSX PET in normal subjects, thus confirming good reproducibility of [^11^C]TMSX PET in the putamen. In this study, we investigated A2ARs in the striata of drug-naïve PD patients and PD patients with dyskinesia, and alteration of A2ARs after antiparkinsonian therapy using [^11^C]TMSX PET.

## Results

**Figure 2:**
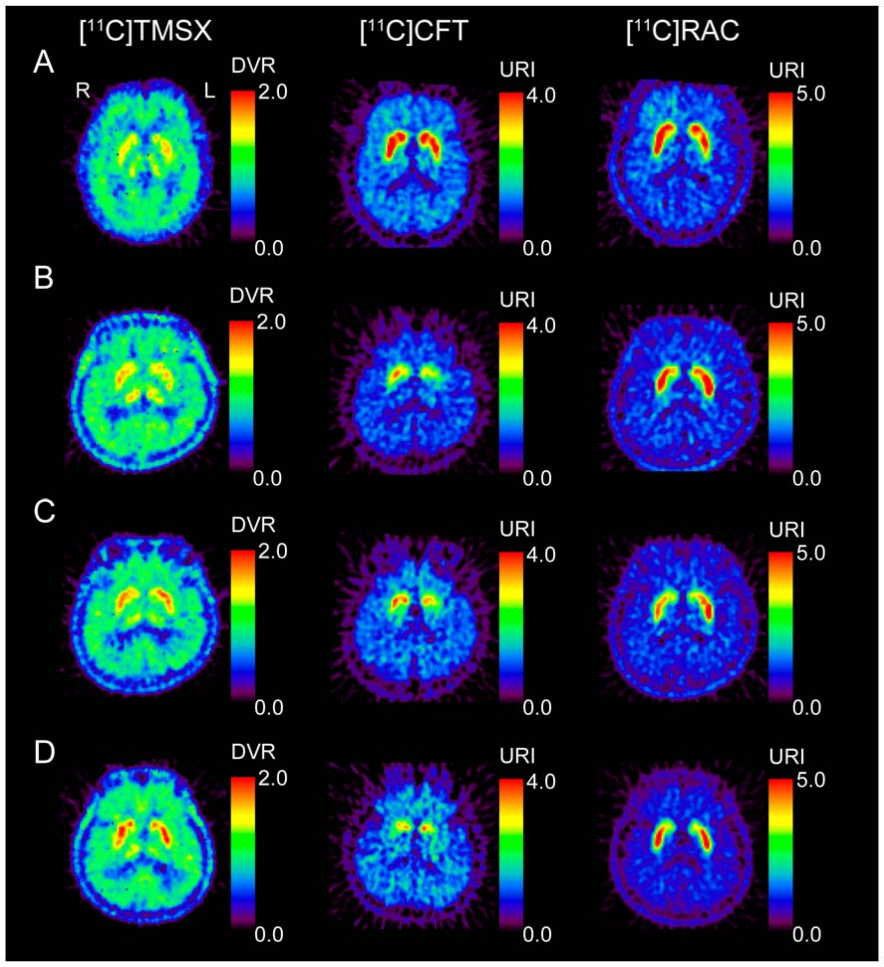
PET images for normal subjects, a drug-naïve patient with Parkinson's disease (PD), and a PD patient with dyskinesia. The normal subjects are a 63-year-old female for [^11^C]TMSX, and a 63-year-old male for [^11^C]CFT and [^11^C]RAC (A). The drug-naïve patient is a 60-year-old male with right-dominant parkinsonism, and underwent two series of PET scans before (B) and after anti-parkinsonian therapy (C). The patients did not developed dyskinesia at a second series of PET scan in the post-therapeutic state. The DVR of [^11^C]TMSX was smaller in the left putamen than in the right and was increased after treatment with anti-parkinsonian therapy for 14 months. The uptake of [^11^C]CFT was reduced in the putamen, especially on the left. The uptake of [^11^C]RAC was preserved in the striatum and was larger in the left putamen than in the right. The relative uptake of [^11^C]CFT and [^11^C]RAC in the putamen was lower in the post-therapeutic state than in the drug-naïve state. The PD patient with dyskinesia is a 65-year-old male with right-dominant parkinsonism (D). His disease duration was 19 years. The DVR of [^11^C]TMSX was increased in the striata. The uptake of [^11^C]CFT was significantly decreased in the putamen.

Figure 2 shows representative PET images for normal subjects, a drug-naïve PD patient before and after anti-parkinsonian therapy, and a PD patinet with dyskinesia.

### Group comparison

There was no significant difference with age among the three groups, i. e. the drug naïve PD patients (five men and four women, mean age 64.6±5.7 years, Table 1), the PD patients with dyskinesia (two men and five women, mean age 65.0±7.3 years, Table 2) and the normal subjects (three men and three women, mean age 60.7±8.5 years). Rate of female was slightly large in the patients with dyskinesia [22], although not significant. In the PD, duration of disease was longer in the patients with dyskinesia (11.1±7.2 years) than the drug naïve patients (2.0±1.2 years, p<0.001, unpaired *t*-test). There was no significant difference with the Unified Parkinson's Disease Rating Scale part III (UPDRS-III) between the drug naïve patients (17.7±13.2) and the patients with dyskinesia (12.6±11.2).

**Table 1:** Demographic and clinical data of drug-naïve patients with Parkinson's disease.

No	Age (years)	Gender	Duration from onset (years)	Symptom	Hoehn & Yahr	UPDRS-III Pre-therapy	Duration of therapy (month)	Antiparkinsonian agents at post-therapy PET	LED (mg)	UPDRS-III Post-therapy
1	72	F	2.0	R>L	1	5	7.0	Levodopa and carbidopa	100	19
2	60	M	1.3	R>L	2	8	14.2	Pramipexole and amantadine	75	6
3	58	F	3.0	R>L	4	41	15.9	Pramipexole, levodopa and carbidopa	450	25
4	68	F	0.5	R>L	2	23	15.9	Pramipexole, amantadine, levodopa and carbidopa	137.5	4
5	64	M	1.2	L>R	1	6	12.9	Pramipexole	150	7
6	56	M	0.7	L>R	2.5	10	20.8	Pramipexole and amantadine	200	21
7	68	M	3.0	L>R	3	18	14.7	Ropinirole, selegiline, droxidopa, levodopa and benserazide	825.5	21
8	71	M	4.0	R>L	1.5	12	–	–	–	–
9	64	F	2.0	L>R	2.5	36	–	–	–	–
Mean±SD	64.6±5.7		2.0±1.2		2.2±1.0	17.7±13.2	14.5±13.2		276.9±272.2	14.7±8.7

UPDRS-III; Unified Parkinson's Disease Rating Scale part III. LED; levodopa-equivalent dose.

**Table 2:** Demographic and clinical data of dyskinesic patients with Parkinson's disease.

No	Age (years)	Gender	Duration from onset (years)	Symptom	Hoehn & Yahr	UPDRS-III	UPDRS-IV	Antiparkinsonian agents	LED (mg)
1	73	F	5	R>L	2	8	5	Pramipexole, Levodopa, carbidopa and entacapone	416.7
2	65	M	19	R>L	3	9	6	Cabergoline, trihexyphenidyl hydrochloride, droxidopa levodopa and carbidopa	667.0
3	66	F	10	R>L	3	7	5	Trihexyphenidyl hydrochloride, zonisamide, levodopa and benserazide hydrochloride	300.0
4	50	F	2	R>L	2	11	3	Pramipexole, levodopa and carbidopa	750.0
5	66	F	9	L>R	3	13	4	Pramipexole, levodopa and carbidopa	225.0
6	65	M	11	L>R	2	3	7	Pramipexole, cabergoline, amantadine, levodopa and carbidopa	983.5
7	70	F	22	L>R	4	37	9	Pramipexole, trihexyphenidyl hydrochloride, amantadine, levodopa and carbidopa	500.0
Mean±SD	65.0±7.3		11.1±7.2		2.7±0.8	12.6±11.2	5.6±2.0		548.9±267.7

UPDRS-III; Unified Parkinson's Disease Rating Scale part III. LED; levodopa-equivalent dose.

The distribution volume ratio (DVR) for [^11^C]TMSX in the bilateral putamen of patients with dyskinesia was larger with that normal controls (p<0.05), although that of drug-naïve patients was comparable with that of normal controls (Table 3). In the head of the caudate nucleus, the DVR of patients with dyskinesia was alightly larger than that of other two groups, although not significant.

**Table 3:** The DVRs for [^11^C]TMSX and URIs for [^11^C]CFT and [^11^C]RAC in the striata of normal controls, drug-naïve patients with Parkinson's disease (PD) and PD patients with dyskinesia.

Radiopharmaceutical	Normal Control	Drug-naïve PD	PD with dyskinesia
Putamen			
[^11^C]TMSX	1.47±0.11	1.48±0.10	1.58±0.15*
[^11^C]CFT	2.67±0.49	1.14±0.33‡	0.65±0.24‡,††
[^11^C]RAC	3.10±0.42	3.43±0.55	2.86±0.70**
Head of the caudate nucleus			
[^11^C]TMSX	1.38±0.08	1.37±0.09	1.44±0.15
[^11^C]CFT	2.52±0.54	1.83±0.35§	1.36±0.39‡,**
[^11^C]RAC	2.70±0.48	2.50±0.37	1.84±0.49‡,§§

Values are mean ± SD (Drug-naïve PD: *n*  =  9; PD with dyskinesia: n  =  7; normal: *n* =  6 for [^11^C]TMSX and *n*  =  8 for [^11^C]CFT and [^11^C]RAC). Binding of [^11^C]TMSX was evaluated as the distribution volume ratio (DVR) and binding of [^11^C]CFT or [^11^C]RAC was expressed as the uptake ratio index (URI).

*p<0.05 (vs normals),

**p<0.05 (vs drug-naïve),

† p<0.005 (vs normal),

†† p<0.005 (vs drug-naïve PD),

§ p<0.0005 (vs normal),

§§ p<0.0005 (vs drug-naïve),

‡ p<0.0001 (vs normal),

‡‡ p<0.0001 (vs drug-naïve PD, Tukey-Kramer *post hoc* test).

In both the bilateral putamen and the head of the caudate nucleus, the uptake ratio index (URI) of [^11^C]2β-carbomethoxy-3β-(4-fluorophenyl) tropane ([^11^C]CFT), a marker for presynaptic dopamine transporter (DAT), in the bilateral putamen was significantly lower in the drug-naïve patients and patients with dyskinesia than in the normal controls (Table 3). The URI of [^11^C]raclopride ([^11^C]RAC), a marker for postsynaptic D2R, in the bilateral striata was lower in the patients with dyskinesia than in the controls and drug-naïve patients (Table 3).

### Asymmetry in drug-naïve state

When we examined the asymmetrical symptoms in the early phase of PD as they relate to the PET images, we found that the DVR for [^11^C]TMSX in the putamen was significantly lower on the more affected side than on the less affected side in the PD patients (Table 4). In contrast, the head of the caudate nucleus showed no significant differences between the more affected side and the less affected side according to DVRs for [^11^C]TMSX.

**Table 4:** The asymmetry in DVRs for [11C]TMSX and URIs for [11C]CFT and [11C]RAC in the striata of drug-naïve patients with Parkinson's disease.

Radiopharmaceutical	More affected side	Less affected side
Putamen		
[^11^C]TMSX	1.46±0.11	1.50±0.10*
[^11^C]CFT	0.98±0.30	1.29±0.29†
[^11^C]RAC	3.59±0.53	3.27±0.56†
Head of the caudate nucleus		
[^11^C]TMSX	1.38±0.10	1.36±0.08
[^11^C]CFT	1.78±0.33	1.89±0.38
[^11^C]RAC	2.52±0.37	2.47±0.39

Values are mean ± SD (PD: *n*  =  9). Binding of [^11^C]TMSX was evaluated as the distribution volume ratio (DVR) and binding of [^11^C]CFT or [^11^C]RAC was expressed as the uptake ratio index (URI).

*p<0.05,

† p<0.005 (paired *t*-test).

The URI for [^11^C]CFT in the putamen was significantly lower on the more affected side than on the less affected side in the drug-naïve patients, and the URI for [^11^C]RAC was significantly higher on the more affected side than on the less affected side (Table 4). In the head of the caudate nucleus, there was no significant difference between the more affected side and the less affected side in the URI for either [^11^C]CFT or [^11^C]RAC.

**Figure 3:**
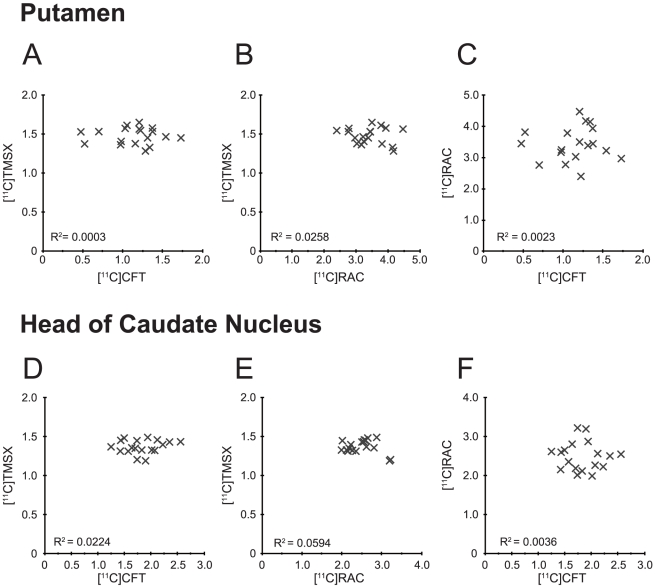
Scattergrams for binding parameters of [^11^C]TMSX, [^11^C]CFT and [^11^C]RAC in the striata of drug-naïve patients. The binding parameter for [^11^C]TMSX was expressed as DVR, and parameters for [^11^C]CFT and [^11^C]RAC were expressed as URI. There was no significant correlation among these variables.

Figure 3 demonstrates the lack of significant correlation among the DVR for [^11^C]TMSX, the URI for [^11^C]CFT and the URI for [^11^C]RAC in the bilateral striata of drug-naïve patients. Specifically, there was no significant correlation among the following variables; the DVR for [^11^C]TMSX versus the URI for [^11^C]CFT (Figure 3A and 3D), the DVR for [^11^C]TMSX versus the URI for [^11^C]RAC (Figure 3B and 3E), and the URI for [^11^C]CFT versus the URI for [^11^C]RAC (Figure 3C and 3F).

### Post-therapeutic state

In the seven PD patients with follow-up studies, the DVR for [^11^C]TMSX in the bilateral putamen was significantly increased in the post-therapeutic state relative to the drug-naïve state (Table 5). The URIs for both [^11^C]CFT and [^11^C]RAC in the bilateral putamen was significantly decreased in the post-therapeutic state than the drug-naïve state.

**Table 5:** Therapeutic change in DVRs for [^11^C]TMSX and URIs for [^11^C]CFT and [^11^C]RAC in the bilateral striata in the seven patients with Parkinson's disease.

Radiopharmaceutical	Drug-naïve state	Post-therapeutic state
Putamen		
[^11^C]TMSX	1.46±0.12	1.50±0.09*
[^11^C]CFT	1.12±0.36	0.88±0.23†
[^11^C]RAC	3.44±0.42	2.76±0.78‡
Head of the caudate nucleus		
[^11^C]TMSX	1.36±0.09	1.37±0.11
[^11^C]CFT	1.74±0.29	1.48±0.23§
[^11^C]RAC	2.56±0.37	2.01±0.52‡

Values are mean ± SD (*n*  =  7). Binding of [^11^C]TMSX was evaluated as the distribution volume ratio (DVR and binding of [^11^C]CFT or [^11^C]RAC was expressed as the uptake ratio index (URI).

*p<0.05,

† p<0.005,

§ p<0.001,

‡ p<0.0005 (paired *t*-test).

In the head of the caudate nucleus bilaterally, the URIs for [^11^C]CFT and [^11^C]RAC were significantly decreased in the post-therapeutic state than the drug-naïve state, but there was no significant difference in the DVR for [^11^C]TMSX.

## Discussion

As previous stidies reported [13], [14], [23], our PET study demonstrates that the putaminal binding ability of A2ARs was increased in the PD patients with dyskinesia, and that there was no significant difference in the striatal binding ability of A2ARs between drug-naïve PD patients and normal controls. However, in drawing attention to the asymmetrical symptoms in drug-naïve PD, our study suggests that A2ARs were asymmetrically down-regulated in the putamen but not in the head of the caudate nucleus. The function of A2ARs is thought to be opposite to that of D2Rs [5]. PET studies using [^11^C]RAC have shown elevated binding of D2Rs in the putamen in early PD [24], [25], [26], due to decreased endogenous dopamine and weak D2R affinity for [^11^C]RAC [27]. However, a postmortem study reported that the density of the D2R binding sites was higher in PD patients than normal controls [28]. Therefore, D2Rs are thought to be up-regulated in the putamen of the patients with advanced PD. Past studies indicated that 80% loss of dopaminergic neurons in the substantia nigra was needed to be developed to symptoms of PD, because such compensatory change of D2Rs is thought to delay the onset of symptoms of PD [29]. We reported on the asymmetrical compensation of the sigma_1_ receptor for the reduction of dopamine in the putamen in early PD [30]. Our study in the drug-naïve state suggested that A2ARs might play an important role in inhibition of asymmetrical parkinsonism in PD along with D2Rs and sigma_1_ receptors.

In the patients with PD, DATs in the putamen were reduced in the diskinesic patients relative to the drug-naïve patients, and reduced in the post-therapeutic state relative to the drug-naïve state. These findings were considered to reflect the progression of PD. The uptake of [^11^C]RAC in the putamen was reduced in the diskinesic patietnts relative to the drug-naïve patients, and reduced in the post-therapeutic state relative to the drug-naïve state. These findings are consistent with the compensatory change of D2Rs. We think that there are two possible reasons. First, [^11^C]RAC may have competed with the D2R agonists and increased endogenous dopamine due to levodopa, thus decreasing the binding of [^11^C]RAC to D2Rs. Second, the antiparkinsonian therapy may have abrogated the compensation of D2R due to the decrease in the dopamine. These factors can affect the binding of [^11^C]RAC in the striata, and it is difficult to detect the up-regulation of D2R using [^11^C]RAC PET in the patients with PD.

Striatal GABAergic medium spiny neurons (MSNs) represent more than 90% of striatal neurons, and A2ARs are highly expressed in the striatopallidal MSNs (indirect pathway) but not in the striatonigral MSNs (direct pathway) [31], [32]. Postmortem studies demonstrated dendritic atrophy of MSNs during advanced PD [33], [34]. Therefore, an alteration in A2AR expression may be involved in the dendritic atrophy in the MSNs.

Our study showed that the binding of [^11^C]TMSX to A2ARs was increased in the putamen in the PD patients with mild dyskinesia. A postmortem study suggested that the messenger ribonucleic acid (mRNA) of A2ARs and [^3^H]SCH-58261-specific binding to A2ARs were increased in the putamen of PD patients with dyskinesia following long-term levodopa therapy [14]. Another study showed that mRNA levels for the A2ARs were elevated in the putamen of levodopa-treated MPTP monkeys compared with controls, although autoradiography with [^3^H]SCH-58261 could not showed significant difference [23]. Our study also showed that the binding of [^11^C]TMSX was increased in the putamen after the antiparkinsonian therapy. The finding may reflect alteration in compensation for the decrease of dopamine in the patients with PD. The drug-naïve patients in this study did not develop dyskinesia by the time of second series of PET scans. Therefore, our study suggests that the increase in putaminal A2ARs after antiparkinsonian therapy preceded the development of dyskinesia in patients with PD. Dyskinesia may be involved in the additional gain of the A2ARs. We may predict the onset of dyskinesia using sequential [^11^C]TMSX PET examination. Moreover, a recent study suggested that dyskinesia may involve not only A2ARs but also A1Rs [35]. Further PET studies with [^11^C]MPDX and [^11^C]TMSX will be needed to clarify this issue.

In conclusion, [^11^C]TMSX PET demonstrated that the distribution of A2ARs was increased in the putamen in PD patients with dyskinesia. A2ARs was asymmetrically down-regulated in the putamen in drug-naïve patients with PD, and the asymmetrical regulation of A2ARs seems to be involved in compensation for the decrease in dopamine. Our study also showed that the A2ARs were increased in the putamen after antiparkinsonian therapy.

## Materials and Methods

### Subjects

We studied nine drug-naïve patients with PD. Table 1 summarizes the clinical profiles of the patients. All patients were right-handed. Their PD diagnoses were based on their medical histories, physical and neurological examinations, laboratory tests, and magnetic resonance imaging (MRI) studies, to rule out other diseases [1], [36], [37]. They had no medical histories of bronchial asthma and did not take theophylline regularly. To confirm early diagnosis of PD, each patient was also examined for DATs and D2Rs by PET using [^11^C]CFT and [^11^C]RAC, respectively. Specifically, we confirmed low binding of [^11^C]CFT and normal or high uptake of [^11^C]RAC in the putamen of all patients [38], [39], [40]. After PET examinations for drug naïve state, we also confirmed that the patients with PD were markedly ameliorated the parkinsonian symptoms by antiparkinsonian therapy, and that their diagnoses of PD remained unchanged in more than two years of observation. The patients did not experience hallucinations or dementia [41]. UPDRS-III was used to evaluate motor disability.

Seven of the nine patients were re-examined with [^11^C]TMSX, [^11^C]CFT and [^11^C]RAC PET 14.5±4.1 months after starting antiparkinsonian therapy (Patients 1–7 in Table 1). The daily levodopa equivalent dose (LED) was calculated as follow: levodopa (mg) + levodopa (mg) ×1/3 (if on entacapone) + levodopa (mg) ×0.02× selegiline (mg) + pramipexole (mg) ×100 + ropinirole (mg) ×16.7 + cabergoline (mg) ×67 + pergolide (mg) ×100 + bromocriptine (mg) ×10. The LED for patients that underwent follow-up studies ranged from 75.0 to 825.5 mg (276.9±272.2 mg) at the time of post-therapeutic PET scanning. No patients developed dyskinesia during the period of this study. The administration of antiparkinsonian agents was not stopped before obtaining the PET scans at the therapeutic state.

We also studied advanced PD patients with mild dyskinesia. Table 2 summarizes the clinical profiles of the patients. All patients were right-handed. The LED for patients ranged from 225.0 to 983.5 mg (548.9±267.7 mg) at the time of PET scanning. The Unified Parkinson's Disease Rating Scale part IV (UPDRS-IV) was used to evaluate drug side effects such as dyskinesia. The dyskinesia of the patients was mild, and they could acquire PET examinations without any trouble caused by undesirable movements during the PET scanning.

For [^11^C]TMSX PET scanning, the control group consisted of six volunteers. For [^11^C]CFT and [^11^C]RAC PET scanning, another control group was used, which consisted of eight volunteers (five men and three women, mean age ± SD, 62.3±6.9 years). The subjects in these control groups were all right-handed. They did not have a history of neurological disease or any abnormalities on physical or neurological examinations. In addition, they did not take medications known to affect brain function and did not have a history of alcoholism. The normal subjects for [^11^C]TMSX PET had no medical histories of bronchial asthma.

The study protocols were approved by the Ethics Committee of the Tokyo Metropolitan Institute of Gerontology. Written informed consent was obtained from all subjects who participated in this study.

### [^11^C]TMSX PET

PET was performed in the Tokyo Metropolitan Institute of Gerontology Positron Medical Center with an SET-2400W PET scanner (Shimadzu Co., Kyoto, Japan) [46]. Image manipulations were carried out on a MacBookPro computer with medical image processing software Dr. View/Linux R2.5 (AJS Inc., Tokyo, Japan) implemented in CentOS 5.4 (The CentOS Project, http://www.centos.org/) and Parallels Desktop 5 (Parallels Holdings, Ltd., Renton, WA, US).

All subjects avoided caffeine consumption over 12 hours before obtaining the [^11^C]TMSX PET scans. The [^11^C]TMSX was prepared as previously described [42]. Specific activity at the time of injection ranged from 40.6 to 119.5 GBq/μmol (69.6±20.8 GBq/μmol). After a transmission scan with a rotating ^68^Ga/^68^Ge line source to correct for the photon attenuation using the attenuation map, a dynamic series of decay-corrected PET data was acquired for 60 minutes using the 2D mode starting at the time of 700 MBq of [^11^C]TMSX injection without arterial blood sampling. A total 27 of frames were taken, including six 10-s frames, three 30-s frames, five 1 min frames, five 2.5 min frames, and eight 5 min frames. All procedures for [^11^C]TMSX PET scanning were performed under dim light to prevent photoisomerization of the [^11^C]TMSX [42], [43].

We generated early images for [^11^C]TMSX PET by summing frames of the dynamic scan from 0 to 10 minutes [44], and we used these images as a reference for placing ROIs on the PET images of dynamic scans. We placed circular ROIs 10 mm in diameter on the PET images over the putamen and the head of the caudate nucleus bilaterally. In addition, we placed ROIs over the frontal lobe, temporal lobe, and occipital lobe to use as reference regions for kinetics analysis. We calculated regional time activity curves in the tissue (tTACs) from the dynamic data and ROIs, and we evaluated binding of [^11^C]TMSX to A2ARs in the striata as a DVR using tTACs, a graphical analysis with the cerebral cortex as the reference region [45]. A kinetics analysis of the tTACs was performed using programs implemented in MATLAB version 7.04 (The Mathworks, Natick, MA, USA).

### [^11^C]CFT and [^11^C]RAC PET

The [^11^C]CFT was prepared as previously described [47]. The specific activity at the time of injection ranged from 10.3 to 300.3 GBq/µmol (51.9±60.1 GBq/µmol). Transmission data were acquired with a rotating ^68^Ga/^68^Ge rod source for attenuation correction. Each subject received an intravenous injection of 300 MBq of [^11^C]CFT. Beginning 75 minutes after the injection, an emission scan was performed using the 3D mode, which lasted 15 minutes.

The [^11^C]RAC was prepared as previously described [48]. The specific activity at the time of injection ranged from 5.7 to 280.0 GBq/µmol (63.1±57.4 GBq/µmol). Transmission data were acquired with a rotating ^68^Ga/^68^Ge rod source for attenuation correction. Each subject received an intravenous injection of 300 MBq of [^11^C]RAC. Beginning 40 minutes after the injection, an emission scan was performed using the 3D mode, which lasted 15 minutes.

Circular regions of interest (ROIs) 10 mm in diameter were positioned on the PET images over the bilateral putamen, the head of the caudate nucleus and the cerebellar hemisphere. For a semi-quantitative analysis of the [^11^C]CFT and [^11^C]RAC PET, we calculated URI in the putamen and the head of the caudate nucleus [49] as follows:



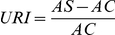



where *AS* is the radioactivity in the ipsilateral putamen or the head of the caudate nucleus, and *AC* is the radioactivity of the bilateral cerebellar hemispheres.

### Statistics

Among the drug naïve PD patients, the PD patients with dyskinesia and the normal subjects, One-way analysis of variance with Tukey-Kramer *post hoc* test were used to compare DVRs for [^11^C]TMSX PET, URIs for [^11^C]CFT and [^11^C]RAC PET, and age. Where only two groups were compared, unpaired *t* test was used. Group difference in gender was calculated with the use of the chi-square test of proportions. In the drug naïve patients, striata from the more affected and the less affected side were compared, and the effect of therapeutic treatment on the striata was evaluated by the paired *t*-test. Regression analyses were used for comparison among binding parameters of [^11^C]TMSX, [^11^C]CFT and [^11^C]RAC. The level of significance was set to p<0.05. Statistical values were computed using the software package JMP version 9.0.0 (SAS Institute Inc., Cary, NC, USA) on a Macintosh computer.
